# Cyclic phosphatidic acid relieves osteoarthritis symptoms

**DOI:** 10.1186/1744-8069-10-52

**Published:** 2014-08-14

**Authors:** Mari Gotoh, Aya Nagano, Ryoko Tsukahara, Hiromu Murofushi, Toshiro Morohoshi, Kazuyuki Otsuka, Kimiko Murakami-Murofushi

**Affiliations:** 1 Endowed Research Division of Human Welfare Sciences, Ochanomizu University, 2-1-1 Ohtsuka, Bunkyo-ku, Tokyo 112-8610, Japan; 2 Science and Education Center, Ochanomizu University, 2-1-1 Ohtsuka, Bunkyo-ku, Tokyo 112-8610, Japan; 3 SANSHO Co. Ltd, 1-2-10 Nihonbashi, Chuo-ku, Tokyo 103-0027, Japan; 4 Osaka Biomedical Professional School, 1-14-30 Shimanouchi, Chuo-ku, Osaka 570-0011, Japan

**Keywords:** Cyclic phosphatidic acid, 2-carba-cyclic phosphatidic acid, Osteoarthritis, Total meniscectomy, Synoviocytes, Chondrocytes, Chonrosarcoma SW1353 cells, Hyaluronic acid, Metalloproteinase

## Abstract

**Background:**

Cyclic phosphatidic acid (cPA) is a naturally occurring phospholipid mediator with a unique cyclic phosphate ring at the *sn*-2 and *sn*-3 positions of its glycerol backbone. Natural cPA and its chemically stabilized cPA derivative, 2-carba-cPA (2ccPA), inhibit chronic and acute inflammation, and 2ccPA attenuates neuropathic pain. Osteoarthritis (OA) is a degenerative disease frequently associated with symptoms such as inflammation and joint pain. Because 2ccPA has obvious antinociceptive activity, we hypothesized that 2ccPA might relieve the pain caused by OA. We aimed to characterize the effects of 2ccPA on the pathogenesis of OA induced by total meniscectomy in the rabbit knee joint.

**Results:**

Intra-articular injection of 2ccPA (twice a week for 42 days) significantly reduced pain and articular swelling. Histopathology showed that 2ccPA suppressed cartilage degeneration in OA. We also examined the effects of 2ccPA on the inflammatory and catabolic responses of human OA synoviocytes and chondrosarcoma SW1353 cells *in vitro*. 2ccPA stimulated synthesis of hyaluronic acid and suppressed production of the metalloproteinases MMP-1, -3, and -13. However, it had no effect on the production of interleukin (IL)-6, an inflammatory cytokine. The suppressive effect of 2ccPA on MMP-1 and -3 production in synoviocytes and on MMP-13 production in SW1353 cells was not mediated by the lysophosphatidic acid receptor, LPA1 receptor (LPA1R).

**Conclusions:**

Our results suggest that 2ccPA significantly reduces the pain response to OA by inducing hyaluronic acid production and suppressing MMP-1, -3, and -13 production in synoviocytes and chondrocytes.

## Background

Cyclic phosphatidic acid (cPA) is a naturally occurring phospholipid mediator that was originally isolated from the myxoamoebae of a true slime mold, *Physarum polycephalum*, in 1992 [1]. Later, cPA was found in mammalian tissues [2,3]. cPA has distinct biological activities; it inhibits autotaxin [4-6], suppresses cancer cell invasion and metastasis [4,7], attenuates ischemia-induced delayed neuronal cell death in rat hippocampal CA1 regions [8,9], and inhibits chronic and acute inflammation-induced C-fiber stimulation, and attenuates neuropathic pain [10]. Therefore, cPA is a promising candidate of therapeutic agent for pain.

**Figure 1:**
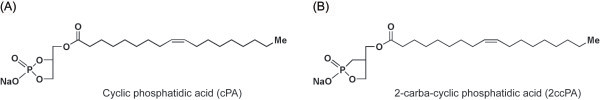
**Structure of cPA and 2ccPA. (A)** Structure of natural occurring cPA 18:1, and **(B)** chemically synthesized its derivative, 2ccPA 18:1, used for the present experiments.

To develop this idea, we synthesized several chemically stabilized derivatives of cPA. cPA has a unique structure comprising of a cyclic phosphate ring at the *sn*-2 and the *sn*-3 positions of the glycerol backbone (Figure 1A), which is required for the biological activities of cPA [11]. In 2-carba-cPA (2ccPA), one of the phosphate oxygens is replaced with a methylene group at the *sn*-2 position (Figure 1B) [4,12]. *In vivo*, tritium labeled 2ccPA ([^3^H]2ccPA) was intravenously injected to rats (30 mg/kg). Then, it has been revealed the half-life of the tritium labeled compound(s) was 81.7 h (unpublished data obtained by Mitsubishi Chemical Medience Corporation). *In vitro*, we have previously shown that cPA 18:1 is stable in neutral-buffered aqueous medium for up to 24 h using liquid chromatography-mass spectroscopy (LC-MS) [4]. The modest drop in cPA concentration over this time frame was not accompanied by a significant increase in LPA levels [4], suggesting that cPA may not be converted into LPA. Furthermore, we investigated the stability of 2ccPA 18:1 in 1 mM phosphate buffered saline using LC-MS/MS, and we revealed that 2ccPA 18:1 was stable for more than 2 weeks at 37°C and it was not converted into LPA (unpublished data). We previously showed that 2ccPA retains many of the biological functions of cPA and that it is a much more potent inhibitor of cancer cell invasion and metastasis and a stronger suppressor of the nociceptive reflex than natural cPA [4,7,10,12].

Osteoarthritis (OA) is a degenerative disease frequently associated with inflammation, joint pain, swelling, and stiffness, leading to significant functional impairment and disability at articular joints [13]. OA is caused by characteristic structural alterations of the joint, including focal degradation of articular cartilage, subchondral bone alterations, and synovitis [14-16]. OA joints are the biological site of inflammation and catabolism. Synovial inflammation likely contributes to the dysregulation of cartilage homeostasis, favoring an imbalance between the catabolic and anabolic activities of chondrocytes in remodeling the cartilage extracellular matrix (ECM) [17-19].

2ccPA inhibites chronic and acute inflammation-induced C-fiber stimulation and attenuates neuropathic pain [10]; we then assessed the ability of 2ccPA to relieve OA-related pain in a rabbit model *in vivo*. In addition, to investigate the molecular mechanisms of 2ccPA in OA-related cells, we examined the effects of 2ccPA on the inflammatory and catabolic responses of human OA synoviocytes and chondrosarcoma SW1353 cells *in vitro*.

## Results and discussion

### Pain assessment

**Figure 2:**
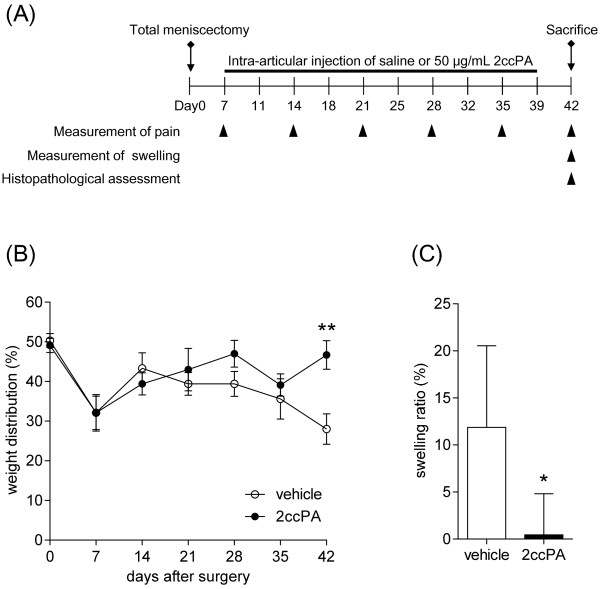
**Effect of 2ccPA on hind-paw weight distribution and articular swelling in a rabbit model of OA. (A)** Experimental schedule. OA was induced by total meniscectomy in rabbits. Vehicle or 2ccPA was intra-articularly injected starting 7 days after surgery. **(B)** The change in weight distribution (%) of the right hind paw from Day 0 to Day 42 in vehicle- (open-circles) and 2ccPA-(closed circles) treated groups. All data are expressed as means ± standard error (SE). **(C)** The percent swelling score was also calculated and expressed as means ± SE. (*P < 0.05 and **P < 0.01 vs. vehicle-treated controls).

Figure 2B shows the change in right hind paw weight distribution (%) from Day 0 (surgery) to Day 42 (sacrifice) for the vehicle- and 2ccPA-treated groups. From Days 7 to 14, both the vehicle- and 2ccPA-treated groups recovered from the surgical stress observed between Days 0 and 7. After Day 14, the weight distribution (%) of the right hind paw of the vehicle-treated group gradually decreased with time, indicating that OA symptoms were induced by meniscectomy. In the 2ccPA-treated group beginning on Day 21, the weight distribution (%) was higher than in the vehicle-treated group. On Day 42, the weight distribution (%) of the 2ccPA-treated group was 1.7-fold higher than that of the vehicle-treated group. These results suggest 2ccPA reduces OA pain in the rabbit meniscectomy model.

### Inflammatory swelling assessment

Figure 2C shows the swelling ratio on Day 42 for the vehicle- and 2ccPA-treated groups. The swelling ratio was 11.87 ± 3.54% in the vehicle-treated group and 0.45 ± 1.78% in the 2ccPA-treated group. These results suggest 2ccPA exerts a constraining influence on the swelling resulting from OA inflammation in the rabbit meniscectomy model. Therefore, we believe 2ccPA is effective for relieving pain and reducing inflammation caused by OA.

### Histopathology

**Figure 3:**
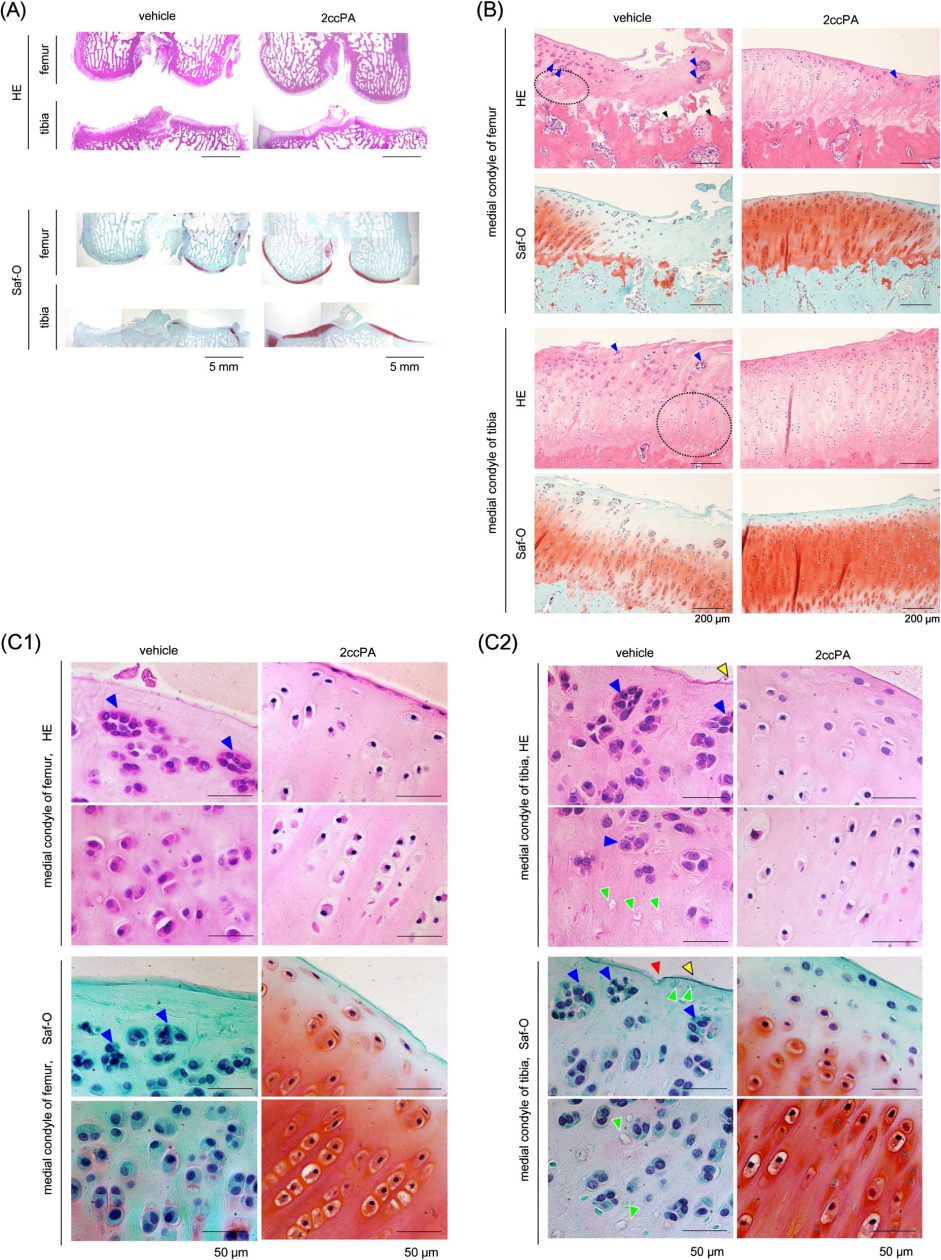
**Histopathology of the medial condyle of the femur and tibia with or without 2ccPA treatment.** Hematoxylin and eosin (HE)- and Safranine (Saf)-O–stained sections of the cartilage of the right medial condyle of the femur and tibia on Day 42 after surgery. Original magnification × 10 **(A)**, ×200 **(B)**, and × 600 **(C1** and **C2)**. Disorganization of chondrocytes (black circles), exposure of subchondral bone (black arrowheads), cluster formation (blue arrowheads), loss of chondrocytes (green arrowheads), loss of the superficial layer (yellow arrowheads), and fissure (red arrowhead) are shown.

We observed remarkable attenuation of pain and articular swelling in 2ccPA-treated animals 6 weeks after surgery (Figure 2B and C). Therefore, we chose Day 42 to evaluate the effects of 2ccPA on OA.Figure 3 shows a representative section of hematoxylin and eosin (HE)- and Safranine-O (Saf-O)-stained sections of cartilage from the right medial condyle of the femur and tibia in the vehicle- and 2ccPA-treated groups on Day 42. Stained-sections revealed typical changes of OA such as disorcanization of chondrocytes (black circles), exposure of subchondral bone (black arrowheads), cluster formation (blue arrowheads), loss of chondrocytes (green arrowheads), loss of the superficial layer (yellow arrowheads), and fissure (red arrowhead).

HE-staining revealed that chondrocyte disorganization in the medial condyle of the femur was significant in the vehicle-treated group. However, chondrocytes were much more ordered in the 2ccPA-treated group than in the vehicle-treated group. Cluster formation was also attenuated in the 2ccPA-treated versus the vehicle-treated group. Saf-O staining showed a significant loss of stainable proteoglycan in the vehicle-treated group. However, proteoglycan loss was imperceptible in the 2ccPA-treated group. These results suggest 2ccPA relieved chondropathy and cartilage degeneration; in addition, the loss of stainable proteoglycan, chondrocyte disorganization, and cluster formation were considerably lower in the 2ccPA-treated group.

Like the medial condyle of the femur, chondrocyte disorganization and cluster formation in the medial condyle of the tibia were significant in the vehicle-treated group; however, these morphologic changes were reduced in the 2ccPA-treated group. Saf-O staining showed that the loss of stainable proteoglycan in the vehicle-treated group was attenuated by 2ccPA.

Other morphologic changes in the medial condyle of the femur and tibia, including loss of the superficial layer, cartilage erosion, and fibrillation and/or fissures were less substantial in the 2ccPA-treated group than in the vehicle-treated group. Due to mechanical friction during meniscectomy in the rabbit OA model, serious cartilage degeneration occurs within a week after surgery and progresses gradually [20,21]. In this study, cartilage degradation induced by meniscectomy was suppressed in the 2ccPA-treated group, suggesting that in the rabbit OA model, 2ccPA may influence pain and catabolic regulation; therefore, 2ccPA provided chondroprotective effects during OA progression.

### Measurement of hyaluronic acid in synoviocyte and chondrosarcoma SW1353 cells in vitro

Synovial inflammation likely contributes to dysregulation of cartilage homeostasis, favoring an imbalance between the catabolic and anabolic activities of the chondrocyte in remodeling cartilage extra cellular matrix (ECM). Cartilage tissue is altered and damaged by inflammatory mediators and degradative enzymes in the synovial fluid of OA [17-19] and the proportion of high-molecular-weight hyaluronic acid decrease while the inflammatory cytokines and ECM-degrading enzyme MMPs increase [17,19,20,22]. The pain and swelling scores in this study demonstrated the antinociceptive and anti-inflammatory effects of 2ccPA administration. Therefore, we investigated the chondroprotective effects of 2ccPA *in vitro* by using synoviocytes and chondrosarcoma cell line SW1353, an *in vitro* model for primary chondrocytes in OA.

**Figure 4:**
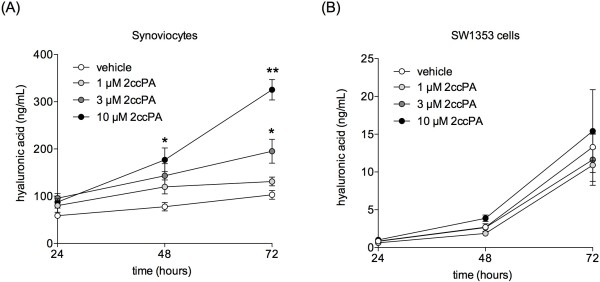
**Effect of 2ccPA on production of hyaluronic acid by synoviocytes and SW1353 cells.** Synoviocytes **(A)** and SW1353 cells **(B)** were cultured with 1, 3, or 10 μM 2ccPA for the time indicated. The concentrations of hyaluronic acid in culture media were determined with ELISA. Data represent the mean ± SE of triplicate independent experiments (*P < 0.05 and **P < 0.01 vs. vehicle-treated controls).

We initially studied the effects of 2ccPA on the production of hyaluronic acid by enzyme-linked immunosorbent assay, ELISA. As shown in Figure 4, the amount of hyaluronic acid increased with incubation time in both cell types and 2ccPA significantly increased hyaluronic acid secretion in a dose-dependent manner in synoviocytes (Figure 4A). Compared with vehicle, 10 μM 2ccPA enhanced hyaluronic acid secretion by 3.2-fold in synoviocytes. On the other hand, SW1353 cells increased production of hyaluronic acid over time, but were unaffected by 2ccPA (Figure 4B). Hyaluronic acid synthetic capacity is much lower in SW1353 cells than in synoviocytes; therefore 2ccPA might not stimulate hyaluronic acid synthesis in SW1353 cells. We suggest 2ccPA stimulates synoviocytes to synthesize hyaluronic acid, thus providing an apparent chondroprotective effect. High-molecular-weight hyaluronic acid inhibits IL-1β-stimulated production of MMP-1, -3, and -13 in chondrocytes [21,23-25]. Therefore, hyaluronic acid induced by 2ccPA may inhibit the production of inflammatory cytokines and MMP-1, -3, and -13 in synoviocytes and SW1353 cells. We investigated the effects of 2ccPA on the inflammatory cytokines and MMPs production of both cells.

### Inflammatory cytokine expression and secretion from IL-1β-stimulated synoviocytes and chondrosarcoma SW1353 cells

**Figure 5:**
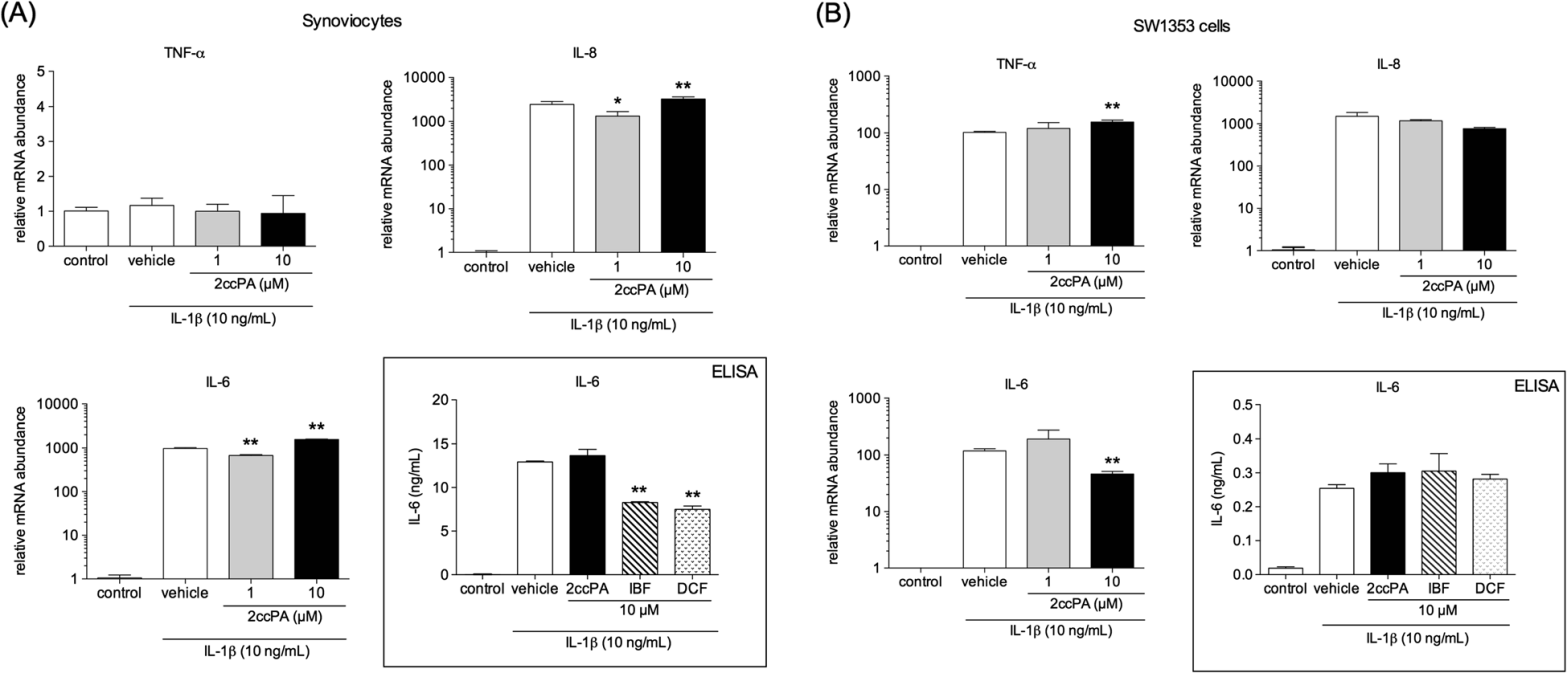
**Effect of 2ccPA on mRNA expression and production of inflammatory cytokines.** To measure mRNA expression, synoviocytes **(A)** and SW1353 cells **(B)** were cultured with 1 or 10 μM 2ccPA for 24 h in the presence or absence of 10 ng/mL IL-1β. mRNA levels of each gene were determined by quantitative real-time PCR. Data represent the mean ± SE of triplicate independent experiments (*P < 0.05 and **P < 0.01 vs. vehicle-treated controls). To measure IL-6 concentrations in the culture media of synoviocytes **(A)** and SW1353 cells **(B)**, cells were cultured with 10 μM 2ccPA, IBF or DCF in the presence of 10 ng/mL IL-1β. After 24 h incubation, culture media were collected, and IL-6 concentrations were determined by ELISA. Data represent the mean ± SE of triplicate independent experiments (*P < 0.05 and **P < 0.01 vs. vehicle-treated controls).

By using quantitative real-time PCR, we investigated transcript expression of inflammatory cytokines IL-1β, IL-6, IL-8, and TNF-α in synoviocytes and SW1353 cells. As shown in Figure 5A, IL-1β treatment for 24 h increased transcript expression of IL-6 and -8, but not TNF-α and IL-1β (data not shown) in synoviocytes. Primary rheumatoid synovial fibroblasts stimulated by IL-1β exhibit increased IL-6 and IL-8 secretion, but TNF-α is not affected [26]. Although the synoviocytes used in this study were obtained from OA patients, the induction of inflammatory cytokines upon stimulation with IL-1β was similar to that of rheumatoid synovial fibroblasts. IL-1β treatment of SW1353 cells induced transcript expression of IL-6, -8, and TNF-α with a maximum at 3 h (data not shown) that was maintained for 24 h (Figure 5B). Expression of IL-1β mRNA did not change over the 24 h assay period (data not shown). The induction of inflammatory cytokines by IL-1β was slightly affected by 2ccPA, and these results suggest that 2ccPA did not have a dramatic effect on inflammatory cytokine production.

It has been reported that the production of inflammatory cytokine IL-6 increases in OA joints and triggers catabolic reactions [16,17]. Then, we performed ELISA to measure the production of IL-6 by both cells in culture media. The amount of IL-6 produced by IL-1β–stimulated synoviocytes and SW1353 cells increased to 12.9 ± 0.2 ng/mL and 0.254 ± 0.011 ng/mL, respectively (Figure 5). On synoviocytes, NSAIDs treatment reduced the inductive effect while 10 μM 2ccPA had no effect on IL-6 production (Figure 5A). On the other hand, NSAIDs and 2ccPA showed negligible effects on IL-6 production in SW1353 cells (Figure 5B).

### MMP-1, -3, and -13 expression and secretion from IL-1β-stimulated synoviocytes and chondrosarcoma SW1353 cells

**Figure 6:**
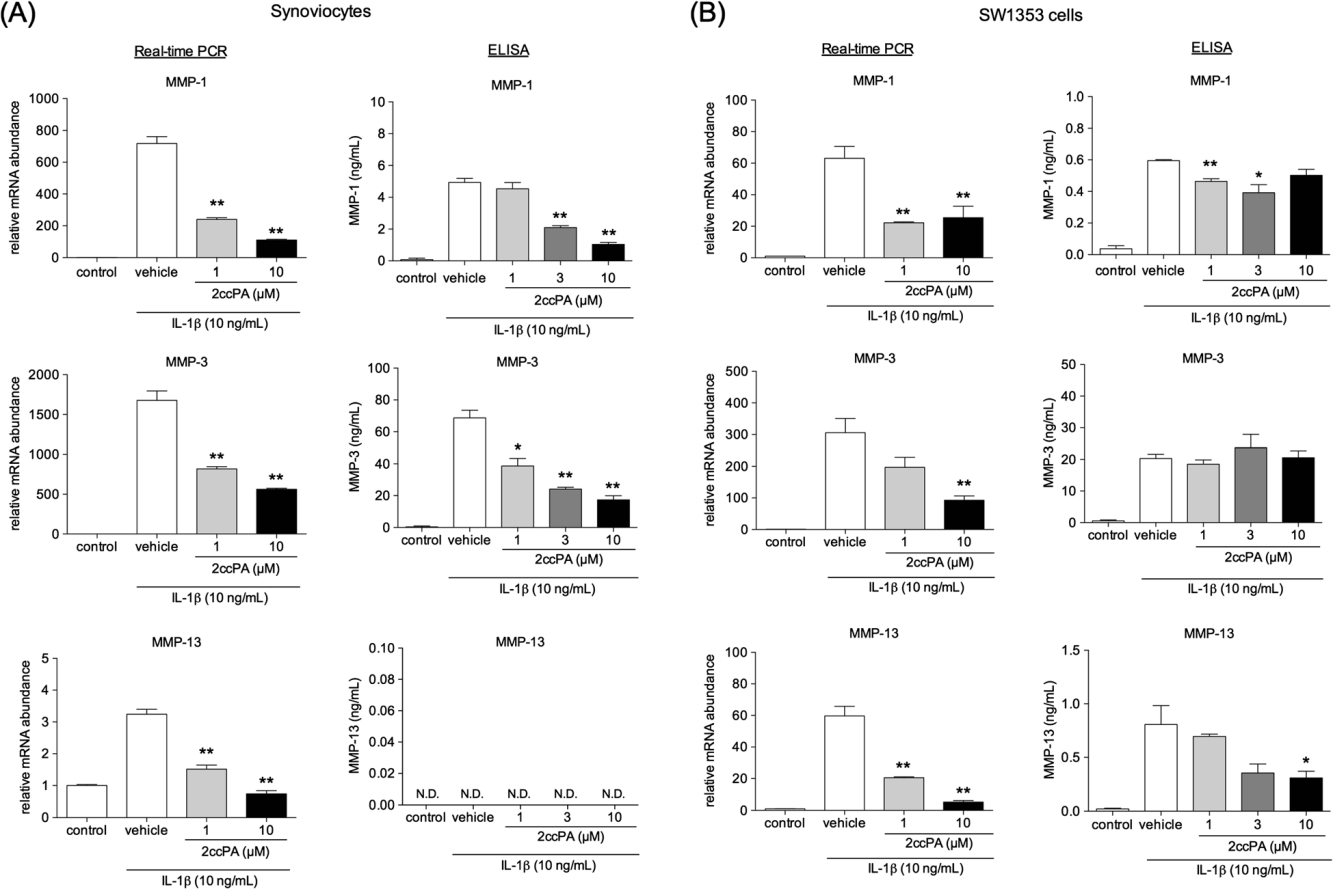
**Effect of 2ccPA on mRNA expression and production of MMP-1, -3, and -13.** To measure mRNA expression, synoviocytes **(A)** and SW1353 cells **(B)** were cultured with 1 or 10 μM 2ccPA for 24 h in the presence or absence of 10 ng/mL IL-1β. mRNA levels were determined by quantitative real-time PCR. Data represent the mean ± SE of triplicate independent experiments (*P < 0.05 and **P < 0.01 vs. vehicle-treated controls). To measure MMPs in the culture media of synoviocytes **(A)** and SW1353 cells **(B)**, cells were cultured with 1, 3, or 10 μM 2ccPA in the presence of 10 ng/mL IL-1β. After 24 h incubation, culture media were collected and the concentration of each MMP was measured by ELISA. Data represent the mean ± SE of triplicate independent experiments (*P < 0.05 and **P < 0.01 vs. vehicle-treated controls). N.D. stands for not detected.

To investigate the effects of 2ccPA on production of ECM-degrading enzymes by synoviocytes and SW1353 cells, we measured MMP-1, -3, and -13, which increase in OA joints and play a role in OA progression [16,18,19]. Transcript expression of MMP-1, -3, and -13 in IL-1β-stimulated synoviocytes and SW1353 cells was assessed by quantitative real-time PCR. IL-1β induced expression of MMP-1, -3, and -13 in both synoviocytes and SW1353 cells after 24 h treatment as shown in Figure 6. The induction of MMPs in synoviocytes and SW1353 cells were significantly reduced by 1, and 10 μM 2ccPA (Figure 6). To investigate the effect of 2ccPA on IL-1β-stimulated MMP-1, -3, and -13 protein expression in synoviocytes and SW1353 cells, we assessed the levels of MMPs secreted into the culture medium.

MMP-1 production increased to 4.9 ± 0.2 ng/mL and 0.60 ± 0.06 ng/mL with IL-1β stimulation in synoviocytes and SW1353 cells, respectively. In synoviocytes, MMP-1 secretion was suppressed dose-dependently by 3 and 10 μM 2ccPA. In SW1353 cells, MMP-1 secretion was suppressed by 1 and 3 μM 2ccPA but dose-dependency was poor. These results suggest that to elicit suppressive function of 2ccPA, it is necessary to choose a certain dose depending on cell types.

MMP-3 production increased to 68.7 ± 4.8 ng/mL and 20.3 ± 1.3 ng/mL with IL-1β stimulation in synoviocytes and SW1353 cells, respectively. MMP-3 production was much higher than that of MMP-1 and -13. In synoviocytes, MMP-3 secretion was suppressed dose-dependently by 2ccPA. In contrast, MMP-3 secretion in SW1353 cells was not affected by 2ccPA treatment.MMP-13 production increased to 0.81 ± 0.18 ng/mL with IL-1β stimulation in SW1353 cells, and the MMP-13 secretion was suppressed by 2ccPA. Although expression of MMP-13 mRNA was observed in synoviocytes (Figure 6A), the amount of protein was not detected.

There are some reports that MMP-1 and -13 degrade collagen and are expressed in synoviocytes and chondrocytes, respectively [18,19]. MMP-3 degrades non-collagen matrix components of the joint and contributes to proteoglycan loss [18,19]; its expression is high in comparison to other MMPs [18]. Our results are consistent with these reports. We suggest that the obvious suppressive effects of 2ccPA on MMP-1 and -3 production in synoviocytes, and on MMP-13 in chondrosarcoma SW1353 cells may offer the appropriate evidence to explain the chondroprotective effect we observed *in vivo* (Figure 3).

### LPA1 receptor function and the suppressive effect of 2ccPA in OA

**Figure 7:**
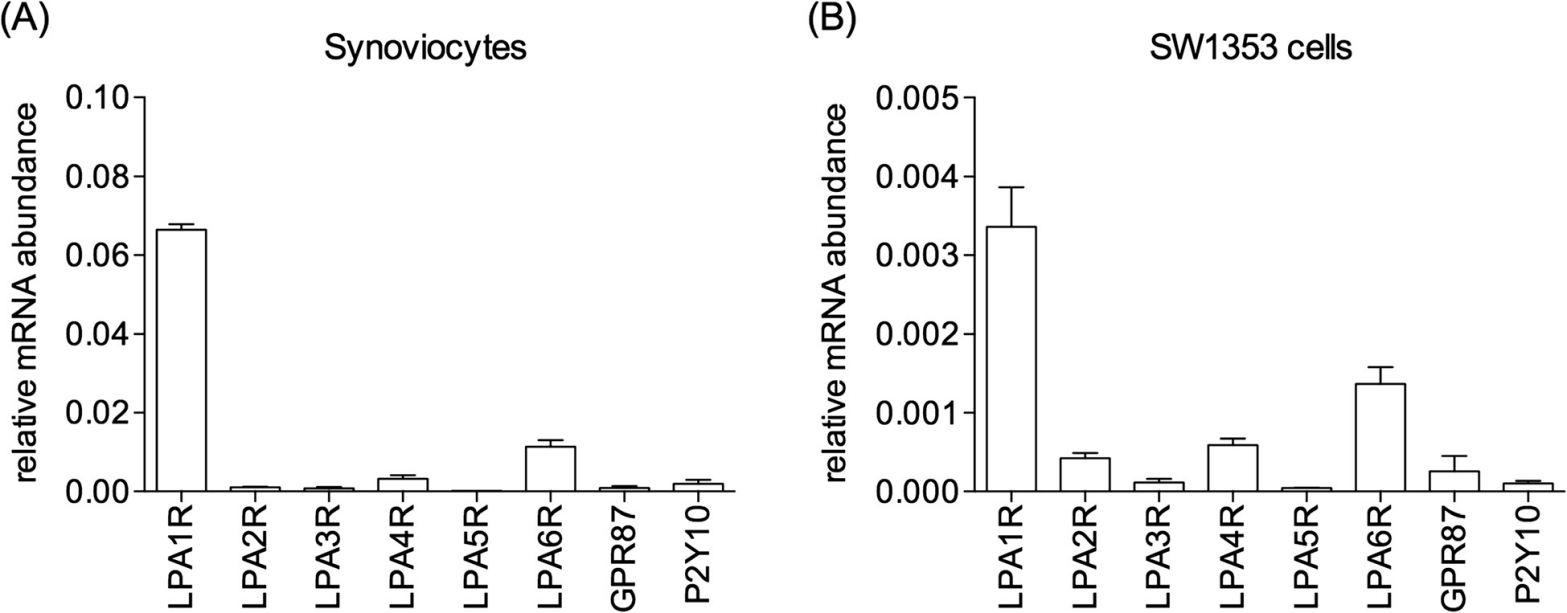
**Expression of LPA receptors in human synoviocytes and SW1353 cells.** Total RNA was extracted and expression of each LPA receptor in synoviocytes **(A)** and SW1353 cells **(B)** was determined by quantitative real-time PCR.

**Figure 8:**
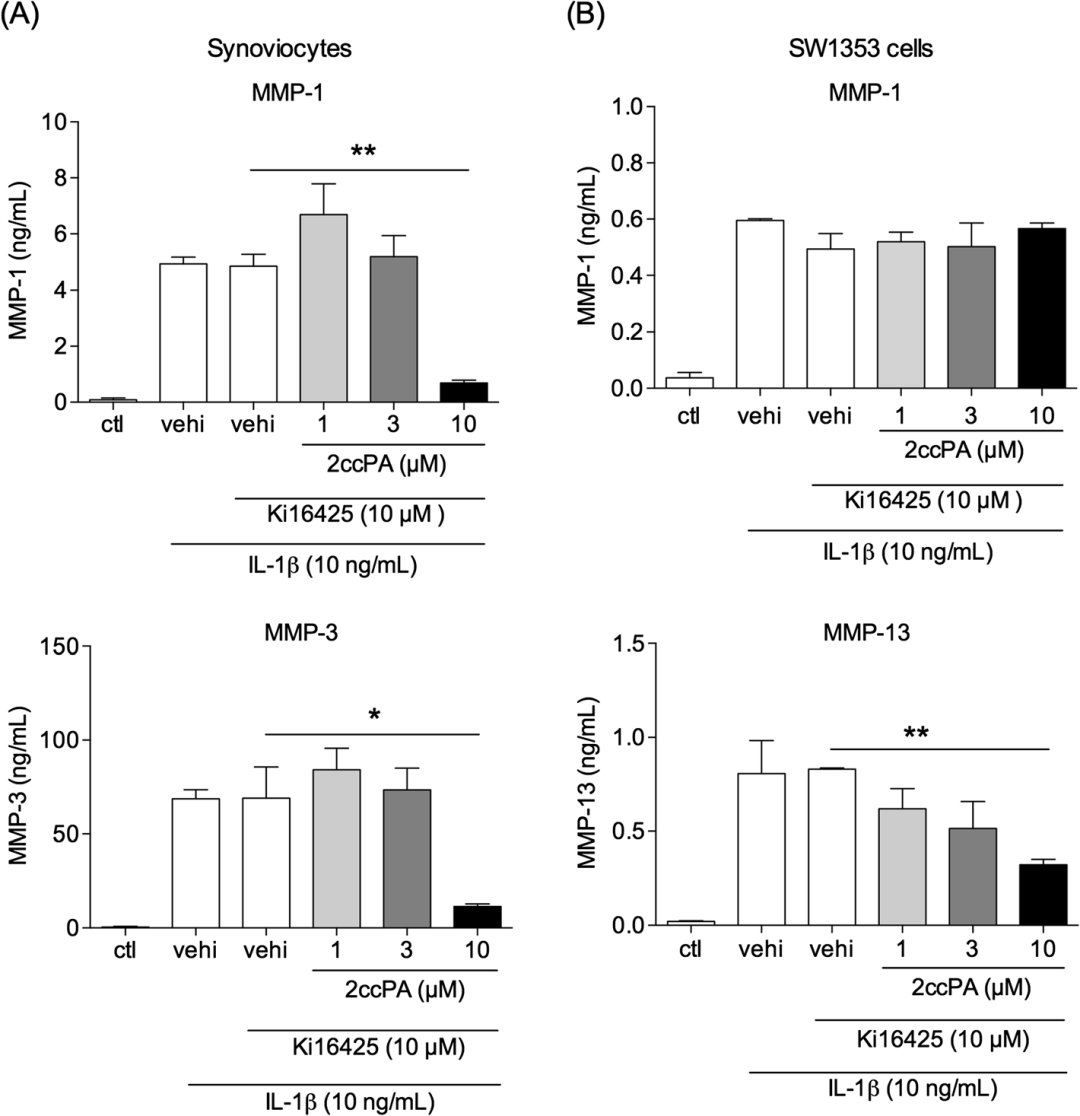
**Effect of Ki16425 on the suppressive function of 2ccPA on MMP-1, -3, and -13 production in synoviocytes and SW1353 cells.** Synoviocytes **(A)** or SW1353 cells **(B)** were pre-incubated with 10 μM Ki16425 for 30 min, then treated with 1, 3, or 10 μM 2ccPA for 24 h in the presence of 10 ng/mL IL-1β. The culture media were collected and the concentration of each MMP was measured by ELISA. Data represent the mean ± SE of triplicate independent experiments (*P < 0.05 and **P < 0.01 vs. vehicle-treated controls).

The pathophysiology of OA appears to be mediated by imbalances between the anabolic and catabolic activity of articular chondrocytes and other joint tissue cells such as synoviocytes [14-19]. Inhibition of the LPA1R has been reported to have therapeutic benefits in Japanese OA [27], although these results were not replicated in a larger sample [28]. Although 2ccPA is an agonist of LPA1R [4], it suppressed OA pathogenesis *in vivo*. We investigated the association of these properties of 2ccPA with the LPA1R signaling pathway and found that synoviocytes and SW1353 cells expressed high levels of LPA1R (Figure 7). In order to examine the involvement of LPA1R in IL-1β-stimulated MMPs production, we tested Ki16425, a selective antagonist for LPA1R and LPA3R. In synoviocytes, Ki16425 did not influence on MMP-1 and -3 production, and it showed no influence on the suppressive effects of 2ccPA on MMP-1 and -3 production (Figure 8A). These results suggest the major receptor for 2ccPA suppression of MMPs in synoviocytes may not be LPA1R. On the other hand, in SW1353 cells, Ki16425 attenuated MMP-1 inhibition by 1 and 3 μM 2ccPA (Figure 8B), but had no effect on MMP-13 production (Figure 8B). Thus, we suggest the major receptor for MMP-13 suppression by 2ccPA in SW1353 cells may not be LPA1R, although this receptor may be involved in MMP-1 suppression. We need to identify the receptor involved in 2ccPA-mediated suppression of MMP-1, -3, and -13 expression hereafter.

Previous studies have suggested that hyaluronic acid reduces MMPs expression in synovial fluid [21,23-25]. Therefore, MMP-1, -3, and -13 suppression by 2ccPA might be due to a 2ccPA-mediated increase in hyaluronic acid synthesis. Further studies are expected to clarify how 2ccPA modulates MMPs expression.

OA causes morbidity, activity limitation, physical disability, excess health care utilization, and reduces health-related quality of life (QOL), especially in people over 60 years old. However, OA management is now limited to the symptomatic treatment of pain and inflammation without reducing joint destruction, which leads to inevitable referral for total joint replacement. Given this unresolved therapeutic need, many challenges remain in the discovery and development of disease-modifying OA drugs (DMOADs) aimed at slowing, halting, or reversing the progression of structural damage of the articular cartilage [29,30]. We believe 2ccPA is a promising DMOAD candidate. The pain-relieving mechanisms of 2ccPA in the pathogenesis of OA are now under investigation.

## Conclusions

Our results suggest 2ccPA significantly reduces the pain response to OA by inducing hyaluronic acid production and suppressing MMP-1, -3, and -13 production in synoviocytes and chondrocytes. These activities might protect chondrocytes from destruction. As a result, pain and inflammatory swelling are relieved. It is strongly suggested that 2ccPA is a promising candidate of DMOAD.

## Materials and methods

### Drug

We used chemically synthesized 2-carba-cPA 18:1 (2ccPA) [4,7]. In the *in vivo* experiments, 2ccPA was dissolved in saline, and saline was used as vehicle. In the *in vitro* experiments, 2ccPA was dissolved in phosphate-buffered saline (PBS) containing 0.1% fatty acid-free bovine serum albumin (BSA), and PBS containing 0.1% BSA solution was used as vehicle.

### OA model

A rabbit model was used to investigate the effects of 2ccPA on the pathogenesis of OA. The design of this animal study was approved by the ethics committee of KAC Corporation (Ethics approval number: 12–0218), a contract research organization (Shiga Japan). All animals were purchased from KITAYAMA LABES Co., Ltd. (Japan), and all animal experiments were performed by KAC Corporation using 11- or 12-week-old male SPF New Zealand white rabbits (*n* = 12, body weight 2.1–2.3 kg). Animals were anesthetized with intravenous (i.v.) pentobarbital (32.4 mg/kg) prior to 1–4% isoflurane followed by subcutaneous infusion of lidocaine (approx. 3 mL) during surgery.

The meniscus of the right leg was totally removed. Briefly, the boundary between the patellar ligament and articular capsule of the right hind leg and the lateral-collateral ligament were dissected. Then, the articular capsule was removed to expose the interior meniscus, and the meniscus was completely removed. Following total meniscectomy of the right knee joint, the rabbits were randomly divided into vehicle- or 2ccPA-treated group. Intra-articular treatment was initiated 7 days after surgery. Vehicle (200 μL saline; Otsuka Pharmaceutical Co., Ltd., Tokyo, Japan) or 50 μg/mL 2ccPA (200 μL) was injected into the joint cavity twice per week over five consecutive weeks (at 7, 11, 14, 18, 21, 25, 28, 32, 35, and 39 days after surgery). The animals were individually caged (48.5 × 30 × 35 cm^3^), received tap water ad libitum, and were fed a standard diet (CR-3, 150 g/day; CLEA, Japan Inc.) throughout the trials. During the experiments, the SPF room temperature was 18°C with unidirectional airflow systems; lighting was provided for 12 h daily (7:00 AM–19:00 PM). The experimental schedule is shown in Figure 2A.

### Measurement of pain

Changes in hind paw weight distribution between the right (OA model) and left (contralateral control) limbs were measured as a pain index [21]. Hind-paw weight distribution was measured once a week (at 7, 14, 21, 28, 32, and 35 days after surgery). The percentage of weight distribution of the right hind paw was calculated with the following equation:

$$\begin{array}{l} \text{Weight}\;\text{distribution}\;\text{of}\;\text{right}\;\text{hind}\;\text{paw}\left(\%\right)=\left(\text{weight}\;\text{of}\;\text{the}\;\text{right}\;\text{leg}/\right)\\ \left(\left[\text{kg}\right]\;\text{weight}\;\text{right}\;+\;\text{left}\;\text{leg}\left[\text{kg}\right]\right)\times 100 \end{array}$$

### Measurement of swelling

At 42 days after meniscectomy, articular swelling was measured with a digital vernier caliper. The maximum widths of the right and left hind paw were measured and recorded. The percentage of swelling was calculated as follows:

$$\begin{array}{l} \text{Swelling}\;\text{ratio}\left(\%\right)=\left[(\text{width}\;\text{of}\;\text{the}\;\text{right}\;\text{knee}\right)\mathrm{mm}-\text{width}\;\text{of}\;\text{the}\;\text{left}\;\text{knee}\left[\text{mm}\right])/\\ \left(\left(\text{width}\;\text{of}\;\text{the}\;\text{right}\;\text{knee}\left[\text{mm}\right]+\text{width}\;\text{of}\;\text{the}\;\text{left}\;\text{knee}\left[\text{mm}\right]\right)\right]\;\times 100 \end{array}$$

### Histopathological assessment of OA

At 42 days after meniscectomy, the rabbits were euthanized by exsanguination immediately after pentobarbital (32.4 mg/kg) administration. The femoral condyle and tibial plateau were resected and immediately fixed in 10% formalin buffer. After decalcification with ethylenediaminetetraacetic acid (EDTA), the samples were cut into 4-μm sections, then stained with HE for general morphology, or Saf-O for proteoglycan, and were observed using a 2.0 MP microscope (H-Micron, Hyogo, Japan, Figure 3A) and an optical microscope BX51TF (OLYMPUS, Tokyo, Japan, Figure 3B, 3C1 and 3C2). Images of 3–6 microscopic fields were incorporated into one image for Figure 3A.

### Cell culture and measurement of hyaluronic acid, IL-6, and MMP-1, -3, and -13 produced by synoviocytes and chondrosarcoma SW1353 cells

All procedures were specifically approved by the ethics committee of Ochanomizu University (Ethics approval number: 24–12) and the National Institute of Biomedical Innovation, Japanese Collection of Research Bioresources Cell Bank (previously Health Science Research Resources Bank, Ethics approval number: 36); the patient gave full written informed consent for tissue donation. Synovial tissue was excised from the knee joint of a 60-year-old female patient with OA during replacement surgery. The patient-derived synoviocytes (Japanese Collection of Research Bioresources Cell Bank, HT91989516, Lot. 07042011) were plated at 1.5 × 10^4^ cells/well with Dulbecco’s modified Eagle medium (DMEM) containing 10% fetal bovine serum (FBS, Life Technologies Corporation, CB) on 12-well plates and incubated overnight at 37°C in humidified 95% air and 5% CO_2_ atmosphere. Chondrosarcoma SW1353 cells were obtained from American Type Culture Collection (ATCC) (no. HTB-94) and were plated at 1.5 × 10^4^ cells/well with DMEM containing 10% FBS on 12-well plates and incubated overnight as well as synoviocytes. The medium was replaced with serum-free DMEM, and the cells were serum-starved for 16 h. To measure hyaluronic acid, 2ccPA was added after serum starvation at final concentrations of 1, 3, and 10 μM and incubated. The culture media were collected at 24, 48, and 72 h, then the concentration of hyaluronic acid was measured by ELISA (R&D Systems, Inc. MN).

To measure IL-6 and MMPs, the cells were treated with either 10 μM of ibuprofen (IBF), diclofenac sodium (DCF) (Wako Pure Chemical Industries, Ltd. Osaka, Japan), or various concentrations of 2ccPA for 24 h in the presence of 10 ng/mL of IL-1β (R&D Systems, Inc.). Culture media were collected at 24 h and the concentrations of IL-6 and MMPs were measured by ELISA kit according to the manufacturer’s instructions (RayBiotech, Inc., GA). For treatment with Ki16425 (Cayman Chemicals, MI), the selective antagonist for LPA1R and LPA3R, the cells were plated at 1.5 × 10^4^ cells/well on 12-well plates and incubated for 30 min with 10 μM of Ki16425 before adding 2ccPA (1, 3, or 10 μM) in the presence of 10 ng/mL IL-1β. Culture media were collected at 24 h and the concentration of each MMP was measured by ELISA kit.

### Quantitative real-time reverse transcription-polymerase chain reaction (RT-PCR)

To quantitate the mRNA levels of inflammatory cytokines (IL-1β, IL-6, IL-8, TNF-α), MMPs (MMP-1, -3, -13), and LPA receptors, real-time RT-PCR was performed with SYBR Premix Ex Taq (Takara Bio, Inc., Shiga, Japan). Total RNA was extracted from cultured synoviocytes and SW1353 cells using ISOGEN reagent (Nippon Gene, Tokyo, Japan) according to the manufacturer’s instructions. cDNA was synthesized with the PrimeScript RT reagent kit (Takara Bio, Inc.). mRNA levels were quantified on a lightCycler 96 system (Roche) instrument. Gene-specific primer sets for IL-1β, IL-6, IL-8, MMP-3, and -13 were described in [31]. Gene-specific primer sets for TNF-α and MMP-1 were described in [32]. Gene-specific primer sets for LPA1R, LPA4R, LPA5R, LPA6R, and p2y10 were described in [7]. The following primer sets were used: GAPDH, 5′-GTGAAGGTCGGAGTCAACG-3′ (F) and 5′-TGAGGTCAATGAAGGGGTC-3′ (R); LPA2R, 5′-GAGGCCAACTCACTGGTCA-3′ (F) and 5′-GGCGCATCTCAGCATCTC-3′ (R); LPA3R, 5′-GAAGCTAATGAAGACGGTGATGA-3′ (F) and 5′-AGCAGGAACCACCTTTTCAC-3′ (R); and GPR87, 5′-AAATCCAGCAGGCAATTCAT-3′ (F) and 5′-CCCTGATGCTCTGGTTATGTT-3′ (R).

The data were calculated based on the Cq values, and the expression of each gene was normalized to GAPDH.

### Statistical analyses

All values are reported as means ± standard error. The data were analyzed using the Student’s *t*-test. A P value less than 0.05 was considered statistically significant.

## Abbreviations

BSA: Bovine serum albumin; ctl: Control; cPA: Cyclic phosphatidic acid; DCF: Diclofenac sodium; DMEM: Dulbecco’s modified Eagle medium; DMOADs: Disease-modifying osteoarthritis drugs; ECM: Extracellular matrix; EDTA: Ethylenediaminetetraacetic acid; ELISA: Enzyme-linked immunosorbent assay; FBS: Fetal bovine serum; HE: Hematoxylin and eosin; IBF: Ibuprofen; IL: Interleukin; i.v.: Intravenous; LC-MS: Liquid chromatography-mass spectroscopy; LPA: Lysophosphatidic acid; LPAR: Lysophosphatidic acid receptor; MMP: Metalloproteinase; OA: Osteoarthritis; PBS: Phosphate-buffered saline; Saf-O: Safranine-O; Vehi: Vehicle; 2ccPA: 2-carba-cyclic phosphatidic acid.

## Competing interests

The authors declare that they have no competing interests.

## Authors’ contributions

MG, AN, RT participated in the experimental designing, collection and analyses of data, and drafted the manuscript. KO was in charge of histological analysis. TM and HM participated in the design of the study and analysis of the data. KMM elaborated a study plan, and was in charge of the overall adjustment of the experimental design and coordination of the whole study. All authors read and approved the final manuscript.
